# Preference for Aggressive End-of-Life Care among Advanced Cancer Patients in Wuhan, China: A Cross-Sectional Study

**DOI:** 10.3390/ijerph17186592

**Published:** 2020-09-10

**Authors:** Jing Liao, Bei Wu, Jing Mao, Ping Ni

**Affiliations:** 1 Tongji Hospital, Tongji Medical College, Huazhong University of Science and Technology, Wuhan 430030, China; m201675378@alumni.hust.edu.cn; 2 School of Nursing, Tongji Medical College, Huazhong University of Science and Technology, Wuhan 430030, China; maojing@hust.edu.cn; 3 Rory Meyers College of Nursing and NYU Aging Incubator, New York University, New York, NY 10010, USA; bw75@nyu.edu

**Keywords:** aggressive end-of-life care, life-sustaining treatments, end-of-life care goal, preference, advanced cancer patients

## Abstract

Life-sustaining treatments (LSTs) and end-of-life (EOL) care’s goal for prolonging one’s life are defined as aggressive EOL care among critically ill patients. They have limited effects and add unnecessary financial burden to advanced cancer patients. A questionnaire survey was conducted to collect information on demographics, disease conditions, preference for LSTs, and goal of EOL care among advanced cancer patients of comprehensive grade-A tertiary hospitals in Wuhan, mainland China. Most patients preferred to accept LSTs when they were in a critical condition, including cardiopulmonary resuscitation (89.9%), mechanical ventilation support (85.7%), nasogastric tube feeding (84.1%), blood transfusion (89.8%), general surgery (87.5%), and hemodialysis (85.8%). Most (88%) preferred prolonging life as the goal of EOL care. Logistic regression showed common influencing factors were participants who completed junior high/high school or below and were financially adequate had higher reference for aggressive EOL care. Patients whose physician had accurately disclosed prognosis; however, showed a decrease trend for aggressive EOL care. Most advanced cancer patients preferred to accept aggressive EOL care. Discussions about prognosis disclosure among physicians and patients should be improved. Education about LSTs’ limitations and comfort-oriented care’s benefits should be promoted among the advanced cancer patients in mainland China.

## 1. Introduction

Aggressive end-of-life (EOL) care has been defined as life-sustaining treatments (LSTs) such as cardiopulmonary resuscitation (CPR), mechanical ventilation support, nasogastric tube feeding, blood transfusion, and general surgery and with the goal of prolonging life among seriously ill patients [1]. Cancer patients have a severe symptom burden and may receive aggressive care at the end of life [2]. However, aggressive EOL care cannot fundamentally improve patients’ survival [3,4,5]; instead, they will add significant burden to the individuals, their family, and the health care system [6,7,8]. On the contrary, for terminally ill patients, an early integration of palliative care is recommended, as it can improves patients’ quality of life (QOL), reduces symptom burden, and subsides EOL care aggressiveness [9]. Advanced cancer patients’ preference for aggressive EOL care were closely related to the intensity of treatments that patients actually received [10,11]. Therefore, to elicit advanced cancer patients’ EOL preferences and explore its related factors was very important to help them avoid futile EOL treatments and refer to palliative care in time.

In the U.S.A., France, Korea, and Japan, relevant studies about preference for aggressive EOL care among cancer patients have been conducted. A retrospective cohort study that enrolled 6111 myeloma decedents found that patients who were transfusion-dependent, on dialysis, or survived for less than one year were more likely to enroll late in hospice and experience aggressive medical care at the end of life in the U.S.A., which suggest meaningful improvements in EOL care [12]. A systematic review concerning cancer from ethnic minority groups found that African Americans preferred aggressive treatment, which may be attributed to inadequate knowledge of EOL care options [13]. A nationwide cohort study conducted in France indicated that older hospitalized adults with cancer with dementia were less likely to receive aggressive cancer treatment near the end of life than those without dementia, such discrepancy raises important ethical questions [4]. A cross-sectional study conducted in Korea found that female cancer patients were more likely to receive CPR, which address the need to improve the planning of EOL care [14]. A retrospective study conducted in Japan found that low-volume hospitals were more likely to provide aggressive EOL care to advanced cancer patients, which may indicate less experience in palliative care or poor quality of EOL care [15].

In the Chinese population, substantial studies done in Taiwan and Hong Kong identified that being male, younger, single, lived in urban areas, with comorbidity, patients whose physician had not disclosed their prognosis accurately or discussed EOL care issues to/with them, patients who did not receive palliative care, and patients who received care in a high-volume hospital were associated with preference for aggressive EOL care [10,16,17]. These studies cannot represent patients in mainland China and be simply implemented as they have different social nature and medical systems [18,19]. In mainland China, few studies concerning about attitudes towards LST have been conducted [20,21]; however, research focusing on the specific issue of aggressive EOL care is lacking, while there has been an urgent need for better EOL care [22].

Therefore, this study aimed to investigate the preference for aggressive EOL care and its associated factors among advanced cancer patients in mainland China.

## 2. Materials and Methods

### 2.1. Study Design and Sample

This study adopted a cross-sectional study design and used a convenience sampling method. All subjects gave their oral informed consent for inclusion before they participated in the study. All participants were informed the aim of the study and their rights to participate in or withdraw from the study at any time. The researchers accompanied the participants throughout the whole interview; if participants had difficulty in understanding the items of the questions, the researchers would explain it in time. The study was conducted in accordance with the Declaration of Helsinki, and the ethical protocol was approved by the institutional review board of the Huazhong University of Science and Technology ([2017] IEC (S054), approved on 23 February 2017). This study was conducted from April 2017 to March 2019.

Advanced cancer patients were recruited by convenience from 2 of the largest comprehensive tertiary hospitals which had patients from entire country in Wuhan, Hubei, mainland China. The recruitment was conducted by a trained research nurse under the help of patients’ doctor in charge. Participants were eligible if (1) they were 18 years old or older, (2) they were diagnosed as III or IV stage cancer in TNM cancer staging system [23], (3) their disease continued to progress and was judged by their oncologist as unresponsive to current anti-cancer treatment, and (4) were capable of providing informed consent. Face-to-face interviews lasting 25-min were conducted by a trained research nurse on weekdays between 9 am and 11 am. The training received by the nurse facilitator included familiarization with the content of the questionnaire, methods for effective communication with patients, methods for communicating with physicians, etc.

### 2.2. Instruments

A questionnaire was developed by the research team based on the current literature [24,25,26], which was reviewed by 5 experts in oncology nursing specialists and geriatric specialists, they judged it to be a valid representation of aggressive EOL care. The questionnaire underwent field pretesting of 30 participants in the oncology department. The Cronbach’s alpha in this study is 0.953. The questionnaire included demographic characteristics, disease information, and preference for LSTs, and goals of EOL care.

#### 2.2.1. Demographic Characteristics

Demographic characteristics included age, gender, marital status, educational level, financial status, and medical insurance. Self-reported financial status was divided into financial sufficiency (making ends meet), and financial strain.

#### 2.2.2. Disease Information

Disease information included cancer type, survival time after the diagnosis, metastasis, whether with comorbidity, whether the physicians informed the patients of the accurate prognosis, and EOL care discussion.

When we asked the patients about their prognosis, we would start with a question, “Can we ask you some questions about your knowledge of your disease?”, and if the patients said, “Yes”, we would go further and ask, “Whether the physician informed you of the prognosis”, and “if so, what the prognosis was?”. Participants were considered to have obtained an accurate prognosis from the physician only if they indicated that the physician had informed them that their disease could not be cured and would continue to deteriorate.

End-of-life care discussions were assessed by asking participants, ‘‘Did the doctor in charge discussed with you about what kind of care you would want to receive if your disease continued to deteriorate, and you were dying?’’ [10].

#### 2.2.3. Preference for LSTs and Goals of EOL Care

LSTs in this study included CPR, mechanical ventilation support, nasogastric tube feeding, blood transfusion, general surgery, and hemodialysis, which were the common treatments in hospitals in China. The response of preference for LSTs included (1) wanted the treatment, or (2) did not want the treatment. Response of goals of EOL care included (1) life-prolonging treatment, and (2) comfort-oriented care.

Participants’ preferences for LSTs and goals of EOL care were elicited by asking the following questions, “If your disease gets worse, and you were in a life-threatening condition, would you like to accept the following LSTs that could only help you sustain your life but cannot recover your health?”, and “If your condition deteriorates, which goal for care would you prefer? Life prolonging or comfort-oriented treatments?” For each LST question, data collectors explained the meaning and pros and cons of LST to the participants. For example, for CPR, it was interpreted as, “A lifesaver that can help the heart beat if the person did not have serious illness before. If people are seriously ill, CPR may not be able to make the heart to beat, but may cause adverse effects as soreness, broken ribs, lungs collapse, etc.”.

### 2.3. Statistical Analysis

The Statistical Package for Social Sciences (SPSS) version 17.0 for Windows (IBM SPSS Statistics, IBM Corporation, Chicago, IL, USA) was used for the quantitative data analyses. Multivariate logistic regression using generalized estimating equation method was used to examine the determinants of preferences for each type of aggressive EOL cares. This method uses a standard error system that explains the correlation in the error terms due to clustering of patients in the same hospital, and adjusts all confounding variables in the model simultaneously [27]. Adjusted odds ratio (AOR) with 95% confidence interval (CI) was estimated for each outcome factor.

## 3. Results

### 3.1. Characteristics of Study Participants

The characteristics of the 775 participants including demographic factors and health status were reported in Table 1. Most of the participants were younger than 60 years old (76.9%), men (54.3%), married (89.8%), and with junior high/high school educational level (57.3%). Participants’ common cancer types were lung (20.4%), breast (12.5%), colon-rectum (8.4%), head and neck (7.6%), and stomach (5.4%) cancer. The median survival time after the diagnosis was 4.0 months.

### 3.2. Preference for Aggressive End-of-Life (EOL) Care

Most participates preferred life prolonging treatment as the goal of EOL care (88%) and preferred to accept CPR (89.9%), mechanical ventilation support (85.7%), nasogastric tube feeding (84.1%), blood transfusion (89.8%), general surgery (87.5%), and hemodialysis (85.8%) separately when life was in a critical condition (Table 1).

### 3.3. Factors Associated with Preference for Aggressive EOL Care among Advanced Cancer Patients

Among demographics factors, age, financial status, and educational level had significant impact on patients’ preference for aggressive EOL care. Patients who were younger than 60 years old were more likely to prefer prolonging life as the goal of EOL care, accepting CPR when life was in a critical condition, and accepting general surgery than patients who were 60 years old or older (Table 2; Table 3). Patients who were in better financial condition were more likely to prefer hemodialysis and mechanical ventilation support (Table 2; Table 3). Patients whose educational level were high school or below were more likely to prefer mechanical ventilation support, nasogastric tube feeding, blood transfusion, and hemodialysis (Table 2; Table 3).

As for disease information factors, patients without comorbidity were more likely to prefer hemodialysis, blood transfusion, mechanical ventilation support, and nasogastric tube feeding than patients with comorbidity (Table 2; Table 3). Patients whose physicians did not disclosed their prognosis accurately to them were more likely to prefer prolonging life as the goal of EOL care, accepting mechanical ventilation support, and hemodialysis (Table 2; Table 3). Patients whose physicians had discussed EOL care with them were more likely to prefer mechanical ventilation support (Table 2; Table 3). Patients whose survival time after the diagnosis were within six months were less likely to prefer CPR when life was in critical condition than beyond six months (Table 2; Table 3).

## 4. Discussion

### 4.1. Preference for Aggressive EOL Care among Advanced Cancer Patients

The prevalence of preference for aggressive EOL care in this study is much higher than previous studies [10,24], which may be due to several factors. Firstly, all participants in this study came from the oncology department of tertiary hospitals that did not have hospice programs [28]. Moreover, LST is generally a trend to use in medical practice in such hospitals [29]. Secondly, less than 20% of patients in our study indicated that their physicians had accurately disclosed prognosis to them, and only 16.6% of patients reported that their physicians had discussed EOL care preferences with them. While patients who lacked of knowledge about palliative care were more likely to choose aggressive EOL care [17]. Thirdly, Chinese public lack knowledge of EOL care, most had never heard of “advance directives” [30]. Fourthly, there is no law regarding EOL care in China, which may also prevent Chinese patients from gaining knowledge of EOL care [22]. Reinforcing education about EOL care, as well as EOL care communication between medical staff and advanced cancer patients can help them avoid futile EOL treatments.

### 4.2. Factors Associated with Preference for Aggressive EOL Care among Advanced Cancer Patients

#### 4.2.1. Age

Patients who were younger than 60 years old were more likely to prefer prolonging life as the goal of EOL care, accepting CPR when life was in a critical condition, and accepting general surgery, which were the same as reported [31,32]. On the contrary, patients who were 60 years old or older were more likely to prefer mechanical ventilation support and nasogastric tube feeding. It can be seen that patients who were younger than 60 years were more likely to prefer higher risky LSTs that require higher physical fitness [33], while older patients were more likely to prefer less risky LSTs that can relieve symptoms quickly [34]. The reason may be that younger patients have stronger will to live [35], while older patients prefer tolerable treatment [36]. However, both higher risky LSTs and less risky LSTs were not suggested, for it has limited effect to advanced cancer patients [4]. Individualized education about aggressive EOL care may help patient know more about the limits of LSTs, which should be implemented among advanced cancer patients in mainland China.

#### 4.2.2. Financial Status

Patients whose financial condition were sufficient/just enough were more likely to prefer hemodialysis and mechanical ventilation support, which were different from a previous study conducted in Taiwan that financial status was not a significant factor [16]. The health insurance in mainland China has limited coverage for medical expenses and prescription drugs [37]. Most of the imported drugs used in the treatment of cancer patients are not covered by health insurance; thus, patients have to pay these drugs out of pocket [38]. However, Taiwan’s national health insurance system had achieved the coverage for comprehensive array of medical services for all of Taiwan’s residents [16]. In our study, only half of the patients have medical insurance, which may influence their decision about aggressive EOL treatment.

#### 4.2.3. With Comorbidity

Patients without comorbidity were more likely to prefer hemodialysis, blood transfusion, mechanical ventilation support, and nasogastric tube feeding, which was contrary to previous study conducted in Taiwan [10]. This may be because patients with comorbidity were in worse health condition [39] while patients with poor health were more likely to prefer less aggressive EOL care [40]. Related education for patients without comorbidity should be prompted, to deepen their understanding of the pros and cons of LSTs and avoid unnecessary treatments.

#### 4.2.4. Prognosis Disclosure

Our study found that patients whose physicians did not disclosed their prognosis to them accurately were more likely to prefer prolonging life as the goal of EOL care, accepting mechanical ventilation support, and hemodialysis as reported. [10] Physicians have been documented as the main source of patients’ prognostic knowledge [41]. Cancer patients who recognized that they were terminally ill preferred symptom-directed EOL care over life-extending therapy [6,42]. Due to the low prevalence of cancer patients’ prognostic disclosure, it is critical to promote rational communication between physicians and patients regarding accurate disclosure of prognosis to help patient make appropriate LST preferences [43].

#### 4.2.5. EOL Care Discussion

Patients whose physicians had discussed EOL care with them were more likely to prefer mechanical ventilation support. On the contrary, patients who were not involved in the discussion of EOL care were more likely to prefer general surgery. This indicates that patients prefer LSTs regardless of whether or not they had EOL care discussion with physicians. However, patients who had undergone an EOL care discussion preferred treatments that were less risky and can relieve symptoms more quickly. EOL care discussion evidently plays a role in preferences [44]. Considering the low prevalence of physician-patient EOL care discussions, it is vital to promote comprehensive communication to help patients make proper EOL care goals.

#### 4.2.6. Survival Time after the Diagnosis

Patients whose post-diagnosis survival time were within six months were less likely to prefer CPR when life was in a critical condition, which was contrary to previous study that patients whose post-diagnosis were within six months were more likely to prefer aggressive EOL care [10].

This discrepancy may due to the lower healthy literacy among the public in mainland China [45]. A cancer diagnosis is devastating to patients, as public almost equate the term with death in mainland China [46]. In addition, median survival after initial diagnosis was four months in this study, which was smaller than 13 months of previous study [10]. Patients diagnosed with cancer within six months have high risk of anxiety and depression [47], patients might be overwhelmed with the rapid course of the disease. Therefore, for patients who have just been diagnosed with advanced cancer in mainland China, they may feel desperate and think they may die soon; thereby, enabling them to choose less aggressive EOL care. Relevant emotional support should be promoted to help them take control of the emotional distress and therefore improve their QOL [48].

#### 4.2.7. Educational Level

Patients whose educational level were high school or lower were more likely to prefer mechanical ventilation support, nasogastric tube feeding, blood transfusion, and hemodialysis, as reported [10]. Less-educated patients may lack sufficient understanding of the pros and cons of LSTs [16]. While, sufficient physician-patient EOL care discussions could help promote patients’ understanding of LSTs’ limitation [49]. Communication between physician and cancer patients need to be strengthened to help less educated patients make appropriate LSTs decision.

## 5. Limitations of the Study

All the sample were collected in two large hospitals in Wuhan, the findings may not be generalizable to all patients in China. We did not interview patients’ caregivers, who may play an important role in patients’ EOL decision. In addition, most participants were relatively young (under 60 years old), which may influence their subjective needs for aggressive treatment.

## 6. Conclusions

Our study showed that over 80% of Chinese advanced cancer patients preferred aggressive EOL care. Patients whose educational level was high school or below, patients’ financial status was adequate, and physicians has disclosed prognosis inaccurately showed high preference for aggressive EOL care. Our study demonstrates the lack of knowledge regarding aggressive EOL among advanced cancer patients. Education about EOL care and LSTs may be needed in order to help patients receiving appropriate care. Early integration of palliative care is also critical to improve the patients’ quality of EOL. In addition, as physicians were reported as main information source of prognosis disclosure, more discussion about prognosis disclosure between patients and physicians are encouraged. Moreover, relevant communication training about EOL care for physicians is needed. This study found some statistical correlations between sociodemographic variables and patients’ aggressive EOL care preferences; however, patients’ decisions may not be fully explained. Therefore, further qualitative studies may be needed to understand patients’ decisions.

## Figures and Tables

**Table 1:** Participants’ demographic and health status characteristics (*n* = 775).

Characteristics	*n*	%
**Demographics**		
Age, years		
18–59	596	76.9
60–84	179	23.1
Gender		
Male	421	54.3
Female	354	45.7
Marital status		
Married	696	89.8
Widowed	20	2.6
Divorced/separated	15	1.9
Single	44	5.7
Educational level		
Primary or below	157	20.3
Junior high/high school	444	57.3
College or above	174	22.5
Financial status		
Not enough	115	14.8
Sufficient/just enough	660	85.2
Whether have medical insurance		
Yes	388	50.1
No	387	49.9
**Disease Information**		
Cancer type		
Lung	158	20.4
Breast	97	12.5
Colon-rectum	65	8.4
Head and neck	59	7.6
Stomach	42	5.4
Liver-pancreas	12	1.5
Other	342	44.1
Survival time after the diagnosis, month		
≤6	513	66.2
7–12	103	13.3
13–24	77	9.9
≥25	82	10.6
Metastasis		
Yes	400	51.6
No	375	48.4
With comorbidity		
Yes	407	52.5
No	368	47.5
Whether the physicians informed you of the accurate prognosis		
Yes	120	15.5
No	655	84.5
EOL care discussion		
Yes	129	16.6
No	646	83.4
**Preference for Aggressive EOL Care**		
Goal of end of life care		
Life-prolonging treatment	682	88
Comfort-oriented care	93	12
CPR		
Yes	697	89.9
No	78	10.1
Mechanical ventilation support		
Yes	664	85.7
No	111	14.3
Nasogastric tube feeding		
Yes	652	84.1
No	123	15.9
Blood transfusion		
Yes	696	89.8
No	79	10.2
General surgery		
Yes	678	87.5
No	97	12.5
Hemodialysis		
Yes	665	85.8
No	110	14.2

**Table 2:** Factors associated with Preferences for aggressive End of Life (EOL) Care: Prolonging Life as a Goal, Cardiopulmonary Resuscitation (CPR) When Life was in a Critical Condition, and Mechanical Ventilation Support.

Characteristic		Participants		Prolonging Life as the Goal of EOL Care				To Accept CPR When Life Was in a Critical Condition				Mechanical Ventilation Support			
				AOR	95%CI		*p*	AOR	95%CI		*p*	AOR	95%CI		*p*
Gender	Male	421	54.3%	1.129	0.887	1.436	0.32	1.092	0.401	2.973	0.86	1.709	0.541	5.400	0.36
	Female	354	45.7%	Ref				Ref				Ref			
Age, y	<60	596	76.9%	1.293	1.197	1.397	<0.001 *	0.614	0.408	0.925	0.020 *	0.771	0.648	0.917	0.003 *
	≥60	179	23.1%	Ref				Ref				Ref			
Marital status	Married	696	89.8%					1.160	0.766	1.755	0.48	0.713	0.106	4.783	0.72
	Widowed	20	2.6%					2.257	0.399	12.762	0.35	0.421	0.083	2.140	0.29
	Divorced/separated	15	1.9%					1.732	0.590	5.084	0.31	1.143	0.402	3.246	0.80
	Single	44	5.7%					Ref				Ref			
Educational level	Primary or below	157	20.3%	1.331	1.067	1.660	0.011 *	0.952	0.910	0.996	0.031 *	2.994	2.262	3.963	<0.001 *
	Junior high/high school	444	57.3%	1.496	0.938	2.384	0.09	1.429	1.157	1.765	0.001 *	1.753	1.551	1.982	<0.001 *
	College or above	174	22.5%	Ref				Ref				Ref			
Financial status	Not enough	115	14.8%	1.327	0.813	2.166	0.25	1.091	0.709	1.678	0.69	0.930	0.921	0.940	<0.001 *
	Sufficient/just enough	660	85.2%	Ref				Ref				Ref			
Whether the physicians informed you of the accurate prognosis	Yes	120	15.5%	0.593	0.526	0.669	<0.001 *	0.756	0.490	1.166	0.20	0.638	0.507	0.801	<0.001 *
	No	655	84.5%	Ref				Ref				Ref			
EOL care discussion	Yes	129	16.6%	0.698	0.299	1.628	0.40	1.153	0.551	2.416	0.70	1.092	1.015	1.176	0.018*
	No	646	83.4%	Ref				Ref				Ref			
Cancer type	Lung	158	20.4%					0.692	0.559	0.858	0.001 *				
	Breast	97	12.5%					0.576	0.113	2.938	0.50				
	Colon-rectum	65	8.4%					0.662	0.354	1.236	0.19				
	Head and neck	59	7.6%					1.369	0.521	3.597	0.52				
	Stomach	42	5.4%					0.582	0.279	1.212	0.14				
	Liver-pancreas	12	1.5%					0.389	0.311	0.487	<0.001 *				
	Other	342	44.1%					Ref							
Survival time after the diagnosis, month	≤6	513	66.2%					0.481	0.317	0.729	0.001 *	0.903	0.696	1.172	0.44
	7–12	103	13.3%					0.561	0.552	0.570	<0.001 *	0.613	0.166	2.259	0.46
	13–24	77	9.9%					1.020	0.782	1.330	0.88	0.792	0.453	1.383	0.41
	≥25	82	10.6%					Ref				Ref			
Metastasis	Yes	400	51.6%	2.094	0.927	4.730	0.07	1.001	0.924	1.084	0.98	1.107	0.672	1.823	0.68
	No	375	48.4%	Ref				Ref				Ref			
With comorbidity	Yes	407	52.5%	1.466	1.035	2.076	0.031*	0.697	0.470	1.031	0.07	0.690	0.489	0.972	0.034 *
	No	368	47.5%	Ref				Ref				Ref			

* means *p* < 0.05. Abbreviations: CI, Confidence interval; AOR, adjusted odds ratio.

**Table 3:** Factors associated with Preferences for aggressive End of Life (EOL) Care: Nasogastric Tube Feeding, Blood Transfusion, General Surgery, and Hemodialysis.

	Nasogastric Tube Feeding					Blood Transfusion				General Surgery				Hemodialysis			
Characteristic		AOR	95%CI		*p*	AOR	95%CI		*p*	AOR	95%CI		*p*	AOR	95%CI		*p*
Gender	Male	2.248	0.788	6.410	0.13	1.195	0.426	3.352	0.73	2.527	0.848	7.525	0.09	1.373	0.789	2.388	0.26
	Female	Ref				Ref				Ref				Ref			
Age, y	<60	0.684	0.642	0.729	<0.001 *	0.769	0.410	1.443	0.41	1.339	1.078	1.663	0.008 *	0.645	0.227	1.829	0.40
	≥60	Ref				Ref				Ref				Ref			
Marital status	Married	0.687	0.141	3.347	0.64	1.149	0.635	2.079	0.64								
	Widowed	0.352	0.117	1.061	0.06	2.021	0.566	7.216	0.27								
	Divorced/separated	1.367	0.718	2.604	0.34	1.635	0.905	2.952	0.10								
	Single	Ref				Ref											
Educational level	Primary or below	2.653	2.207	3.189	<0.001 *	1.382	1.323	1.444	<0.001 *	1.223	0.957	1.563	0.10	2.704	2.045	3.575	<0.001 *
	Junior high/high school	1.917	1.916	1.918	<0.001 *	1.858	1.609	2.145	<0.001 *	1.121	0.739	1.699	0.59	2.227	1.927	2.573	<0.001 *
	College or above	Ref				Ref				Ref				Ref			
Financial status	Not enough	1.345	0.796	2.271	0.26	1.225	0.815	1.841	0.33	1.040	0.525	2.061	0.91	0.752	0.736	0.767	<0.001 *
	Sufficient/just enough	Ref				Ref				Ref				Ref			
Whether the physicians informed you of the accurate prognosis	Yes	0.785	0.594	1.038	0.09	0.869	0.591	1.278	0.47	0.642	0.285	1.445	0.28	0.626	0.403	0.974	0.038 *
	No	Ref				Ref				Ref				Ref			
EOL care discussion	Yes	0.935	0.818	1.068	0.32	0.975	0.516	1.840	0.93	0.835	0.698	0.999	0.049 *	2.378	0.782	7.229	0.12
	No	Ref				Ref				Ref				Ref			
Cancer type	Lung	1.538	1.238	1.910	0.000 *	0.543	0.361	0.817	0.003 *								
	Breast	2.212	0.615	7.954	0.22	0.520	0.079	3.423	0.49								
	Colon-rectum	1.572	0.745	3.317	0.23	0.657	0.357	1.207	0.17								
	Head and neck	2.302	1.010	5.247	0.047 *	2.420	0.827	7.079	0.10								
	Stomach	1.062	0.337	3.342	0.91	0.758	0.303	1.893	0.55								
	Liver-pancreas	0.497	0.395	0.627	<0.001 *	0.320	0.284	0.361	<0.001 *								
	Other	Ref				Ref											
Survival time after the diagnosis, month	≤6	0.984	0.793	1.222	0.88	0.841	0.580	1.218	0.35					1.115	0.968	1.284	0.13
	7–12	0.859	0.259	2.852	0.80	0.978	0.812	1.178	0.81					0.878	0.295	2.619	0.81
	13–24	1.105	0.706	1.731	0.66	1.598	0.973	2.625	0.06					1.076	0.627	1.846	0.79
	≥25	Ref				Ref								Ref			
Metastasis	Yes	1.093	0.743	1.608	0.65	1.081	0.989	1.181	0.08	0.921	0.468	1.810	0.81	1.175	0.825	1.672	0.37
	No	Ref				Ref				Ref				Ref			
With comorbidity	Yes	0.587	0.371	0.929	0.023 *	0.594	0.494	0.715	<0.001 *	0.748	0.333	1.682	0.48	0.695	0.552	0.876	0.002 *
	No	Ref				Ref				Ref				Ref			

* means *p* < 0.05. Abbreviations: CI, Confidence interval; AOR, adjusted odds ratio.
